# Overall Key Performance Indicator to Optimizing Operation of High-Pressure Homogenizers for a Reliable Quantification of Intracellular Components in *Pichia pastoris*

**DOI:** 10.3389/fbioe.2015.00107

**Published:** 2015-08-03

**Authors:** Xavier Garcia-Ortega, Cecilia Reyes, José Luis Montesinos, Francisco Valero

**Affiliations:** ^1^Bioprocess Engineering and Applied Biocatalysis Group, Departament d’Enginyeria Química, Escola d’Enginyeria, Universitat Autònoma de Barcelona, Bellaterra, Spain

**Keywords:** cell disruption, high-pressure homogenization, intracellular component, optimization, overall key performance indicator, *Pichia pastoris*

## Abstract

The most commonly used cell disruption procedures may present lack of reproducibility, which introduces significant errors in the quantification of intracellular components. In this work, an approach consisting in the definition of an overall key performance indicator (KPI) was implemented for a lab scale high-pressure homogenizer (HPH) in order to determine the disruption settings that allow the reliable quantification of a wide sort of intracellular components. This innovative KPI was based on the combination of three independent reporting indicators: decrease of absorbance, release of total protein, and release of alkaline phosphatase activity. The yeast *Pichia pastoris* growing on methanol was selected as model microorganism due to it presents an important widening of the cell wall needing more severe methods and operating conditions than *Escherichia coli* and S*accharomyces cerevisiae*. From the outcome of the reporting indicators, the cell disruption efficiency achieved using HPH was about fourfold higher than other lab standard cell disruption methodologies, such bead milling cell permeabilization. This approach was also applied to a pilot plant scale HPH validating the methodology in a scale-up of the disruption process. This innovative non-complex approach developed to evaluate the efficacy of a disruption procedure or equipment can be easily applied to optimize the most common disruption processes, in order to reach not only reliable quantification but also recovery of intracellular components from cell factories of interest.

## Introduction

*Pichia pastoris* has been widely used as cell factory in the last years (Potvin et al., 2012). The cytoplasm of yeast cells is a rich source of bio-products, such proteins, cytoplasmic enzymes, or polysaccharides valuable in biotechnology, pharmacology, and food industry (Liu et al., 2013b). The quantitative recovery of the intracellular compounds is determined by the disruption processes, which may affect the stability and the biological activity of the desired product. Thus, the selection of a suitable cell disruption method to recover these compounds and its reliable quantification is very important (Liu et al., 2013a). Disruption can be considered a general term that describes different processes related to cellular disintegration that range from slight release of internal metabolites to full cell breakage (Spiden et al., 2013). The efficiency of cell disruption implies selective and complete release of the product to achieve a high recovery of the target products, reduced contaminants, and minimal micronization of cell debris (Harrison, 1991; Middelberg, 1995; Balasundaram et al., 2009).

The existence of cell wall in the yeast cells requires that the disruption and release of intracellular components destructs the strength-provide components of the wall, in the case of yeasts, namely glucans (Liu et al., 2013b). The basic structural components of the yeast cell wall were identified by Smith et al. (2000). In the case of *P. pastoris* disruption procedures, the use of methanol in the cultivation has a relevant impact on the cell wall in comparison with other carbon sources, such glycerol or glucose. An important widening of cell wall thickness of *P. pastoris* cells growing on methanol was described by Canales et al. (1998), which rather increased twice. Furthermore, after the observation of the difficulties to obtain reproducible and reliable results for disruption methods for *P. pastoris* cells grown on methanol, one can consider that more severe methods and operating conditions than the standard reported for *Escherichia coli* or *Saccharomyces cerevisiae* are needed (Balasundaram et al., 2009).

Several methods for disruption of microbial cells are described in the literature; the most commonly used are summarized in some reviews (Harrison, 1991; Middelberg, 1995; Geciova et al., 2002). Most of them have been applied in *Pichia*: sonication (Lin et al., 2007), bead milling (Pfeffer et al., 2012; Grillitsch et al., 2014), enzymatic and chemical lysis (Naglak and Henry, 1990; Boettner et al., 2002), cell permeabilization (Shepard et al., 2002; Lenassi Zupan et al., 2004), and high-pressure homogenization (Johnson et al., 2003; Tam et al., 2012; Gurramkonda et al., 2013). The last one is described as the most used for large scale cell disruption processes in the biopharmaceutical manufacturing industry (Fonseca and Cabral, 2002; Lin et al., 2007).

Despite cell disruption is a field widely studied, among the works published in the literature, there is not agreement about the reporting indicators that should be chosen for its study. The selection of reliable and simple indicators to measure the degree of cellular disruption is a key point to assess the efficiency of the disruption methods. These indicators must not be degraded in the rupture processes and released from the cell consistently through different cycles. Usually, the measure of the target protein released is the best method to quantify the efficiency of the cell rupture (Middelberg, 1995). However, in some cases, the release of other intracellular components can be used as an alternative to determine the extent of cell disruption. Direct and indirect measurements indicating the cellular disruption degree, using *S. cerevisiae* as model, have been recently reviewed concluding that different indicators provide different information to monitor the level of disruption (Spiden et al., 2013). Thus, the combination of different reporting indicators in a single parameter of the disruption efficiency is useful to integrate the information given by each indicator, which can facilitate the efficacy study of the process.

Accurate quantification of intracellular proteins, enzymatic activities, and metabolites is basic to carry out research in biochemistry and biotechnology, from determining cellular components to metabolomics and systems biology studies. The reliability of the target component quantification relies on whether the cell disruption process is efficient and reproducible due that the use of non-optimized procedures may not allow to achieve the complete release of the elements that are being studied, which could lead to important errors in the determination of this cellular components. Thus, it is essential that the cell disruption procedures used always assures efficacy and reproducibility. Furthermore, since *P. pastoris* is commonly used as a recombinant production cell factory, the reliable recovery and quantification of the intracellular product is of capital interest to completely evaluate the efficiency of the bioprocess (Gogate and Pandit, 2008; Pfeffer et al., 2011).

In this sense, the aim of this work is to present a methodology to determine the disruption settings that allows the reliable quantification of a wide sort of intracellular components. This approach can be applied to the most used cell disruption processes. Specifically, the work aims to characterize and optimize the working conditions of a lab scale *HPH* using *P. pastoris* suspensions. This study was performed through the definition of an overall key performance indicator (*KPI*) based on the combination of the following reporting indicators: decrease of absorbance, release of total protein, and release of alkaline phosphatase activity. The reporting indicators have been selected among the main parameters used in other references from the literature, those being preferred, which are simple, rapid, and do not require expensive equipment (Middelberg, 1995). Since this *KPI* aims to be applicable to study different disruption processes, it is important that the reporting indicators selected are not specific for particular organisms, as could be some intracellular small molecules or metabolites. The usefulness of the methodology has been confirmed for a bigger process scale using a pilot plant *HPH*. Finally, the optimal results for *HPH* were compared with other commonly used disruption methodologies.

## Materials and Methods

### Microorganism

Suspensions of a wild-type X-33 *P. pastoris* strain growing on mixed feeds of glucose and methanol were obtained from steady state chemostat cultures. The cultivations were set at a D of 0.09 h^−1^ by feeding a defined growth medium containing 50 g⋅L^−1^ of glucose/methanol mixture (80% glucose/20% methanol, w/w) as a carbon source, dissolved oxygen levels were kept at a minimum of 15% of air saturation, pH was controlled at 5, and temperature at 25°C. More details about the cultivation conditions can be found elsewhere (Jordà et al., 2012).

### Cell disruption methodologies

Prior to the disruption processes, the cell suspensions were always cleaned three times by centrifugation and resuspension in fresh *PBS*. The clean suspensions were vortexed vigorously for homogenizing the samples and dispersing any cellular aggregates. All the samples were kept on ice within the disruption steps in order to avoid the activity of the endogenous proteases. To discard the cell debris after the cell rupture procedures, the disrupted samples were always clarified by centrifugation (4200 × *g*, 4°C, 15 min).

#### High-Pressure Homogenization

High-pressure homogenizer (HPH) is the most employed method for the disruption of microbial cells in large scale bioprocesses. The cell suspension is released at high pressure through a specially designed valve assembly, where the cells are disrupted as a consequence of the different forces produced by the interaction between the fluid and the solid walls of the valve (Kleinig and Middelberg, 1998).

The *One-Shot Cell Disrupter* (Constant Systems Ltd., Warwickshire, UK) was used at lab scale, being 8 mL the volume of the disruption samples. In previous studies, different pressures were compared in order to optimize the method. Two kbars and up to three passes were selected as the best working pressure and maximal number of passes (*N*) for *P. pastoris* as a compromise between the efficacy of disruption and the amount of foams produced during the disruption passes, which introduces lack of reproducibility and uncertainty in the process (Van Hee et al., 2004).

Additionally, at pilot plant scale, the homogenizer used was the *TS Series Cabinet Disruption System* (Constant Systems Ltd., Warwickshire, UK), being 250 mL the volume of the disruption samples. The working pressure was 2.7 kbar, the highest of the equipment, because better disruption results were observed without a substantial increase in foaming production. It is accordingly to its exponential dependence previously referred for *HPH* (Middelberg, 1995).

#### Bead Milling

It is a standard cell disruption in which the intracellular cell components are released after the cell cracking caused by the collisions between beads and cells (Ricci-Silva et al., 2000). The performed procedure using glass beads (Sigma-Aldrich G-9268, 425–600 μm) was adapted from the literature (Sreekrishna et al., 1989). The disruption mixture composed by equal volumes of cell suspension with equivalent initial *OD* (*OD*_0_) of 25 and glass beads were vortexed for 1 min 10 times, each followed by 1 min on ice.

#### Cell Permeabilization

It is an alternative method for the recovery of intracellular proteins from yeast and other microbial cells and organisms, which aims avoiding the common disadvantages of high-pressure homogenization, such as the own complex background of the host producer and mechanical stresses that may affect the recovery and biological activity of the target protein (Somkuti et al., 1998). The used protocol was adapted from a previous published work (Shepard et al., 2002). This is based on suspending and incubating the cells in an aqueous solution containing *N,N*-dimethyltetradecylamine. The working conditions were as follows: 5 g⋅L^−1^ of *N,N*-dimethyltetradecylamine, equivalent initial OD (*OD*_0_) of 9, and incubation time of 15 h.

### Analytical methods

#### Optical Density

Optical density (OD) at 600 nm is commonly used to determine cellular concentration. *OD* measures, in absorbance units (*AU*). It can be easily converted to dry cell weight values (*DCW*, g⋅L^−1^) using the following conversion factor: OD (AU) × 0.2 = DCW (g⋅L^−1^) (Resina et al., 2005). Additionally, in the presented work, *OD* has been used as a direct measure of the cell integrity of the samples; hence, a relative decrease of *OD* was associated to the proportion of cells disrupted (Spiden et al., 2013). All spectrophotometric analyses were taken in triplicate.

#### Total Protein Released

Total protein released (*TPR*) was considered a suitable indirect performance indicator to be correlated to disruption efficiency (Middelberg, 1995). It was determined by Bradford assay, which was performed with Coomassie Plus™ Protein Assay Reagent (Pierce, Rockford, IL, USA) using a bovine albumin as standard. *TPR* assays were taken in triplicate and the relative SD (*RSD*) was about 5%.

#### Alkaline Phosphatase Activity Released

As an intracellular enzyme, the alkaline phosphatase activity released (*APAR*) gives not only an indication of the protein released but also the preservation of enzymatic activity, which was considered as a reliable indirect performance index (Melendres et al., 1993). The protocol was adapted from the literature (Berstine et al., 1973). Alkaline phosphatase was assayed at pH 10.0 using *p*-nitrophenyl phosphate as substrate, incubation time at 37°C was 20 min, after which time absorbance was measured at 410 nm. *APAR* assays were taken in triplicate and the *RSD* was about 6%.

### Data analysis

The *OD* decrease (*ODD*) was determined as a normalized quotient between the pre-homogenized (*OD*_0_), and the post-homogenized (*OD*_H_) values (Eq. 1):

(1) $$\text{ODD}=1-\frac{\text{O}{\text{D}}_{\text{H}}}{\text{O}{\text{D}}_{0}}$$

In the evaluation of the disruption efficacy, the effect of the initial *OD* was one of the variables studied. Since this parameter is directly related to the total amount of biomass that will be disrupted, *TPR* and *APAR* will be affected by this variable. Thus, in order to be able to compare disruption results between samples with different biomass content, these performance indicators were always normalized with the pre-homogenized *OD* of the samples, and hence, using the specific form; specific *TPR* (mg⋅OD^−1^⋅L^−1^); specific *APAR* (AU⋅OD^−1^⋅L^−1^).

**Figure 1:**
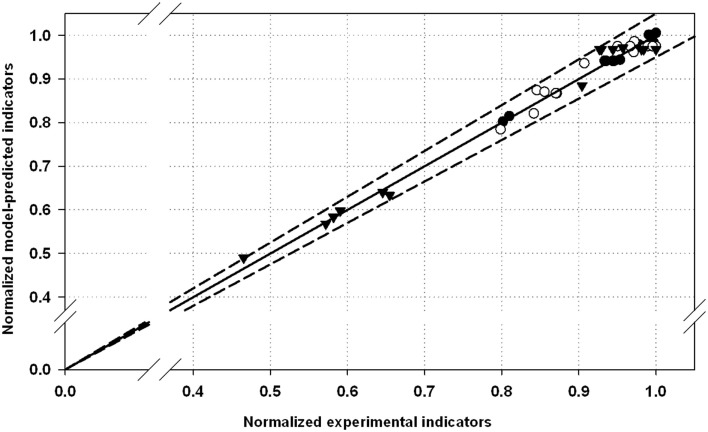
**Normalized parity plot for the selected performance indicators with 5% of error: (•), optical density decrease (*ODD*); (▾), total protein released (*TPR*); (∘), alkaline phosphatase activity released (*APAR*)**. Maximal values for *ODD*, *TPR*, and *APAR* were 88.3%, 85 mg⋅OD^−1^⋅L^−1^, and 0.27 AU⋅OD^−1^⋅L^−1^, respectively.

In the parity plot depicted in Figure 1, all the performance indicators values (*Y*_k_) were normalized with the corresponding maximal value observed at the best process conditions (*Y*_k,max_). In this way, the disruption indicators were scaled and shown together in the same plot.

(2) $$\bar{Y_{\text{k}}}=\frac{Y_{\text{k}}}{Y_{\text{k}},\max}$$

### Experimental set-up and statistical analysis for the design of experiments

The effect of *OD*_0_ and *N* on *ODD*, *TPR*, and *APAR* was studied by means of a Box–Wilson Central Composite Design (*CCD*) and response surface methodology (*RSM*). The *CCD* performed was a face-centered design (*CCF*), which was composed by 13 experiment based in two variables having 3 levels each and 5 central points for replication. The *OD*_0_ and *N* range were 20–100 and 1–3, respectively. These ranges were selected from results obtained in preliminary disruption experiments. The empirical response surfaces were built from the values of *ODD*, specific *TPR*, and specific *APAR*. The data results were fit to the empirical model expressed at Eq. 3:

(3) $$Y_{\text{k}}=\beta_{\text{0,k}}+\sum \beta_{\text{i,k}}\cdot X_{\text{i}}+\sum \beta_{\text{ii,k}}\cdot X_{\text{i}}^{2}+\sum \beta_{\text{ij,k}}\cdot X_{\text{i}}\cdot X_{\text{j}}$$

where, *X*_1_ = OD_0_, *X*_2_ = N; *k* = 1 for *ODD*, *k* = 2 for *TPR*, and *k* = 3 for *APAR*.

The Sigma Plot statistical package (SigmaPlot 11.0; Systat Software, Inc., Chicago, IL, USA) was used in order to perform the statistical analysis and fit the response surfaces. The quality of the fit is expressed by the coefficient of determination *R*^2^ obtained by regression analysis. Additionally, a lack of fit test was performed in order to compare the experimental error to the prediction error. The overall significance of the model was determined by analysis of variance (*ANOVA*) *F*-test, whereas the significance of each coefficient was determined by the corresponding *t*-test. SE of the estimate (*SEE*, %) was also calculated for all three models to test their estimation capabilities.

## Results and Discussion

### Characterization of the *HPH* by means of *DoE*

Design of experiments (*DoE*) and the *RSM* were used to describe the effects of *OD*_0_ and *N* in the cell disruption of *P. pastoris* using a *HPH*. *ODD*, *TPR*, and *APAR* measures were used as quantitative indicators of the disruption degree. This work seeks to take into account more than one reporting parameter for the disruption efficiency evaluation. The experimental results presented were used to estimate the coefficients of the quadratic polynomial equation described in the Eq. 3 (Table 1).

**Table 1:** **Experimental set-up for a *CCF* design for two factors, matrix design and response**.

Experiment	Initial *OD*	Number of passes	*OD* decrease (%)	Total protein released (mg⋅OD^−1^⋅L^−1^)	*AP* activity released (AU⋅OD^−1^⋅L^−1^)
1	20 (−1)	1 (−1)	71.0	49.1	0.218
2	20 (−1)	2 (0)	84.5	55.5	0.231
3	20 (−1)	3 (+1)	87.8	56.2	0.230
4	100 (+1)	1 (−1)	77.2	40.0	0.238
5	100 (+1)	2 (0)	86.6	50.0	0.265
6	100 (+1)	3 (+1)	88.6	50.7	0.265
7	60 (0)	1 (−1)	71.7	77.7	0.234
8	60 (0)	2 (0)	83.8	79.8	0.264
9	60 (0)	2 (0)	82.8	85.9	0.260
10	60 (0)	2 (0)	83.6	79.6	0.273
11	60 (0)	2 (0)	82.9	84.3	0.270
12	60 (0)	2 (0)	82.9	84.6	0.272
13	60 (0)	3 (+1)	88.3	82.2	0.248

Table 2 outlines the estimated coefficients determined for the models. The *ANOVA F*-test associated *p*-value can be used as indicator of the statistical significance of the coefficients on the response. Coefficients without significance are those with *p-*value >0.05. The high values of *R*^2^ and low values of *SEE*, always below 4%, point out a proper goodness of the fit for all the models.

**Table 2:** **Estimated coefficients of the models and ANOVA analysis for the three disruption models in which the experimental results were fitted to the following equation:**
$$Y_{\text{k}}=\beta_{\text{0,k}}+\sum \beta_{\text{i,k}}\cdot X_{\text{i}}+\sum \beta_{\text{ii,k}}\cdot X_{\text{i}}^{2}+\sum \beta_{\text{ij,k}}\cdot X_{\text{i}}\cdot X_{\text{j}},$$
where, *i* = 1 for *OD*_0_, *i* = 2 for *N*; *k* = 1 for *ODD*, *k* = 2 for *TPR*, and *k* = 3 for *APAR*.

	*OD* decrease *ODD*-model			Total protein released *TPR*-model			*AP* activity released *APAR*-model		
	Coefficient	*t*-Value	*p*-Value	Coefficient	*t*-Value	*p*-Value	Coefficient	*t*-Value	*p*-Value
β_0_	51.05	22.97	<0.0001	−0.32	−0.05	0.9594	0.132	6.7717	0.0003
β_1_	−0.03	−0.61	0.5618	2.19	18.43	<0.0001	1.10E-03	2.9067	0.0228
β_2_	24.51	11.72	<0.0001	16.01	2.80	0.0266	0.081	4.4537	0.003
β_11_	0.00	3.54	0.0094	−0.02	−22.72	<0.0001	−7.63E-06	−2.8456	0.0248
β_22_	−3.76	−7.62	0.0001	−3.41	−2.53	0.039	−0.020	−4.558	0.0026
β_12_	−0.03	−3.29	0.0133	0.02	0.82	0.4408	9.88E-05	1.1063	0.3051
*R*^2^	0.9882			0.9900			0.9210
SEE *(%)*	0.99			3.32			2.86

Additionally, a parity plot including all the experimental and model-predicted data is used to present graphically the estimation capabilities of the models (Figure 1). All the experimental points are within the range 5% of error of the fitted model. This also confirms the robustness of the models estimating disruption efficacy in terms of the performance indexes studied.

**Figure 2:**
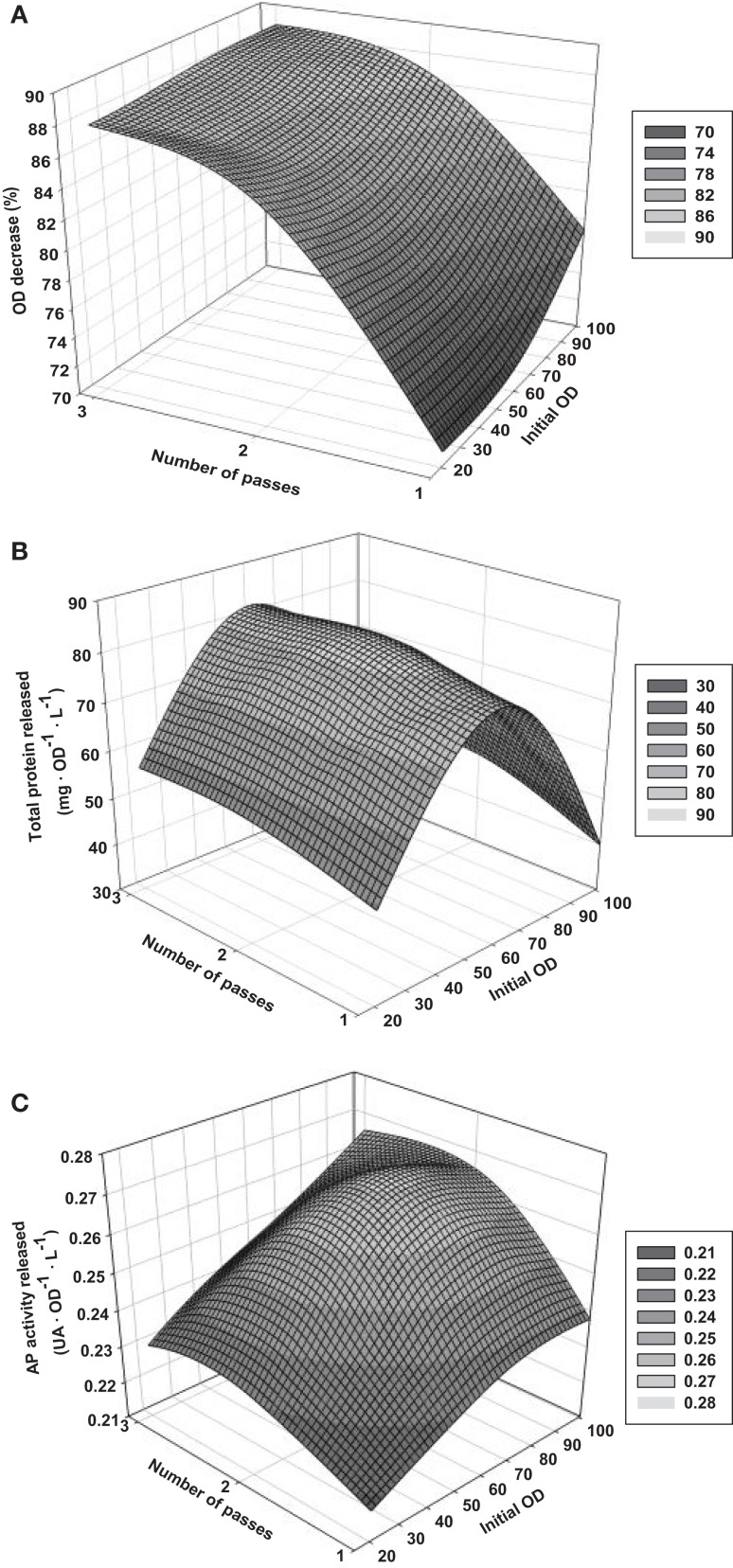
**Response surface graphs based on the results of the *DoE* performed**. **(A)** Optical density decrease (*ODD*); **(B)** total protein released (*TPR*); **(C)** *AP* activity released (*APAR*).

3-D graphs show the effects of the key selected variables in the responses (Figure 2). For the *ODD*-model, *OD*_0_ does not have a clear influence in the response, so the cell concentration of the samples does not influence the loss of cell integrity within the range studied. In contrast, *N* seems to affect the outcome. The higher number of passes, the better result is. However, the difference between two and three passes is slight, what could be related to a plateau effect on the cell rupture phenomenon with increasing *N*. In the case of the *TPR*-model, the *OD*_0_ causes a strong effect in the outcome resulting to a maximal response for intermediate values of initial *OD*. The cell breaking processes that occurs with intermediate cell concentrations seems to be optimal to recover the maximal amount of total protein. *N* does not affect significantly, and hence, one single pass through the HPH is enough to let it out most of the total protein that can be released. For the *APAR*-model, the differences in the response using different conditions are clearly slighter. However, a double plateau effect in both studied variables can be observed, so medium and high *OD*_0_ and *N* leads to high responses of the reporting parameters. Since *TPR* and *APAR* are indirect measures, the results obtained are not only due to cell rupture but also to other processes that can degrade the proteins and may have an important influence on the results. Physical and chemical effects, as well as the action of the proteases of the host cell are considered as the main causes of this degradation. Similarly, it is important to bear in mind that these parameters are also conditioned by the foaming formation during homogenization, what could lead to an inaccurate quantification of the parameters (Van Hee et al., 2004; Tam et al., 2012). Normally, these effects occur on processes aiming to recover the maximal amount of protein as a target product, so it is important that models also take it into account. This fact is corroborated by the slightly higher *SEE* obtained for *TPR* and *APAR* models in comparison with the *ODD*-model.

### Identifying the optimal conditions for *HPH*

According to the results shown and discussed in the previous section, different reporting parameters must be taken into account for analyzing accurately the efficiency of the disruption procedures. Using a lab scale *HPH*, three models have been defined in order to maximize the *ODD*, *TPR*, and *APAR* with two operational variables, *OD*_0_ and *N*. Nevertheless, since different operational conditions must be used to achieve optimal results of the diverse performance indicators, it is of great interest to define an overall performance indicator that can be stated as a global *KPI*. Thus, this *KPI* is used as a global quantification parameter of disruption efficiency and it is calculated as follows:

(4) $$\text{KPI=}\alpha_{\text{1}}\cdot \bar{Y_{1}}+\alpha_{\text{2}}\cdot \bar{Y_{2}}+\alpha_{\text{3}}\cdot \bar{Y_{3}}$$

where $\bar{Y_{k}}$ is the normalized *Y*_k_ calculated dividing by its maximal value obtained *Y*_k,max_; α_k_ is a weighting factor, being ∑ α_k_ = 1. 0; *k* = 1 for *ODD*, *k* = 2 for *TPR*, and *k* = 3 for *APAR*.

From the results obtained after evaluating the effect of the different variables with the *KPI* (Table 3), it is shown that the *N* value that maximizes the *KPI* is 2 in any of the analyzed cases. Nevertheless, the *OD*_0_ optimal values vary between 60 and 80.

**Table 3:** **Maximal overall performance indicator (*KPI*_max_) obtained with a different set of weighting factors and their corresponding number of passes and initial *OD***.

	α_1_	α_2_	α_3_	Number of passes	Initial OD	KPI_max_
1	0.3	0.6	0.1	2	62	0.975
2	0	0.5	0.5	2	61	0.989
3	0.5	0	0.5	2	80	0.971
4	0.5	0.5	0	2	65	0.964
5	1/3	1/3	1/3	2	65	0.970

Among the different weighting criteria, the one that does not take into account the *TPR* is the option that results into a higher difference in the optimal *OD*_0_. This fact leads to conclude that the *TPR* is the key parameter that causes the major differences in the disruption efficiency. However, since this work seeks to take into account more than one reporting parameter, the final selected criterion was the one that took into consideration all the indicators but giving to the *TPR* weighting factor as a higher value.

Consequently, the optimal working conditions of the studied *HPH* have been defined as: working pressure, 2 kbar; *OD*_0_, 60; *N*, two passes. These settings are close to the operational conditions proposed for different commercial *HPH* disrupting bacteria and yeast (Bury et al., 2001; Spiden et al., 2013). Nevertheless, as a significant novelty, the recommended conditions given in the presented work has been determined as optimal through the use of *DoE* and an overall *KPI* based on the combination of simple cell disruption indicators. These process conditions are clearly more advantageous than those suggested for *P. pastoris* disruption by Tam et al. (2012) in which the *N* value proposed is 20, resulting into lower overall disruption efficiencies. Although other authors that published previous works in the field concluded that cell concentration does not have an important effect on the disruption efficiency (Brookman, 1974; Middelberg, 1995; Siddiqi et al., 1997; Van Hee et al., 2004; Tam et al., 2012), revising accurately their results on figures, slight differences were observed. The mentioned differences in the results due to the cell concentration are in the same order of magnitude that the described in the present work. Since the aim of this work is to achieve and assure a very accurate, reliable, and reproducible cell disruption procedure; consequently, it has been concluded that the effect of the cell concentration is a significant factor that must be taken into account for optimizing the performance of a HPH.

### Comparison among the alternative disruption methodologies

**Figure 3:**
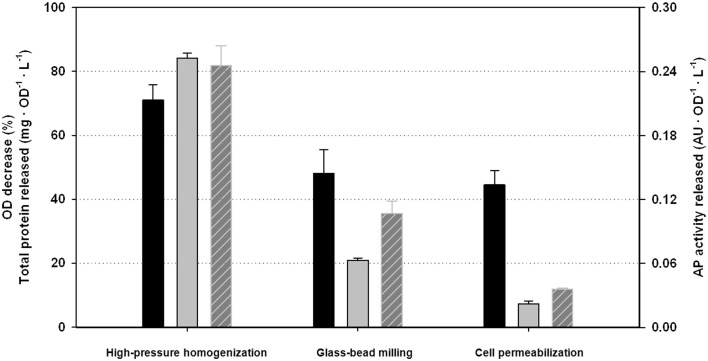
**Comparison of the performance indicators among the alternative disruption methods studied: black, optical density decrease (*ODD*); gray, total protein released (*TPR*); with stripes, *AP* activity released (*APAR*)**.

In order to compare the performance of the *HPH* with some common alternative disruption procedures, bead milling and cell permeabilization were also carried out with the same samples of *P. pastoris*. The previously used quantitative indicators; *ODD*, *TPR*, and *APAR* were also considered as reporting parameters of the disruption efficiency. Results obtained for each procedure are summarized in Figure 3. The operating conditions for the *HPH* were the optimal determined in the previous section. For the other disruption methods, incubation time, number of passes (*N*), and cell and reagent concentrations (*OD*_0_*; R*) were selected following a heuristic procedure and adapted protocols, as described in the Section “Materials and Methods” (data not shown).

As can be stated from all three single performance indexes, results obtained with the *HPH* were clearly better than using the other alternative procedures. *TPR* and *APAR* results using *HPH* were about fourfold higher than using other methodologies. *ODD* results were also significantly higher, at least 50% better. Thus, one can conclude that studying intracellular components, the results obtained with *HPH* will be significantly more consistent and reliable than using other common methods. These results are in accordance with the literature comparing different methods for cell disruption. The use of *HPH* is preferred due to the higher disruption efficiencies obtained and the possibility to scale-up the processes However, the higher cost of the HPH equipment and maintenance are important drawbacks to be taken into account in comparison with other procedures (Geciova et al., 2002; Balasundaram et al., 2009).

From the presented results, it is also important to point out that the profile of the different performance indicators studied is certainly different among the different disruption methodologies. This fact reinforces the need to consider more than one indicator in order to analyze accurately the efficiency of any disruption processes.

### Working conditions comparison between *HPH* at lab and pilot plant scale

In order to compare the performance parameters at a bigger scale, similar working conditions were evaluated for an equivalent *HPH* at pilot plant scale but with operating pressure 2.7 kbar, as previously justified in Section “Materials and Methods.” Cell disruption procedures for two different *OD*_0_, 60 and 100, were performed. The *N* range studied was between one and three passes. The disruption efficiency obtained for OD_0_ = 60 was significantly higher than those for OD_0_ = 100 (data not shown). Consequently, OD_0_ = 60 was selected for the comparison between lab and pilot plant scale *HPH*. The results are presented in Table 4.

**Table 4:** **Comparison table of the performance indicators for the *HPH* at different scales using samples at *OD*_0_ = 60**.

High-pressure homogenizer	*N*	*OD* decrease (%)	SD	Total protein released (mg⋅OD^−1^⋅L^−1^)	SD	*AP* activity released (AU⋅OD^−1^⋅L^−1^)	SD
Lab scale	1	71.7	2.8	77.7	1.1	0.234	0.013
Pilot plant scale	1	79.8	1.2	55.7	0.7	0.181	0.010
Lab scale	2	83.2	2.3	83.1	2.1	0.271	0.005
Pilot plant scale	2	88.2	1.5	60.2	2.2	0.154	0.003
Lab scale	3	88.3	1.7	82.2	1.5	0.248	0.009
Pilot plant scale	3	91.4	1.8	62.1	1.9	0.129	0.003

*SD, standard deviation*.

In terms of *ODD*, the efficacy of the pilot plant scale is slightly better, especially in the first passes. However, the efficiency decreases significantly for both *TPR* and *APAR*. Longer disruption (residence) times for the pilot plant *HPH*, as well as different geometry of the equipment could be feasible reasons for this fact. The decrease of *APAR* in pilot plant scale could be related with proteolysis activity of the endogenous proteases during the longer disruption times.

Afterwards, using the criteria based on the *KPI* and selecting the same weighting factors that in the lab scale (detailed in a previous section), the optimal working conditions at pilot plant scale were determined. These were working pressure, 2.7 kbar; *OD*_0_, 60; *N*, three passes. Since a substantial difference was observed in the disruption performance parameters either using two or three passes, it has been conclude that for this pilot plant *HPH* working with three passes is more effective.

## Conclusion

A *DoE* was conducted to study the effect on the disruption of *OD*_0_ and *N* in a lab scale *HPH*. Three different performance indicators were selected for evaluating the cell disruption degree: *ODD*, *TPR*, and *APAR*. The optimal working conditions of the *HPH* at lab scale were determined by means of the definition of an overall *KPI*, because of the need to consider different indicators for analyzing accurately the efficiency of the disruption processes. Thus, results obtained led to the following optimal operational conditions: 2 kbar; *OD*_0_, 60; *N*, two passes. This disruption method was compared with other commonly used procedures, bead milling and cell permeabilization, showing a disruption efficiency significantly higher for all the reporting parameters studied. These differences were up to fourfold higher in *HPH* for *TPR* and *APAR*.

Finally, the developed approach was also applied to a pilot plant scale *HPH* obtaining similar results for the *ODD*. Nevertheless, an important decrease were observed in the *TPR* and *APAR* indicators, what could be caused by the effect of the endogenous proteases, accompanied by longer residence times and different geometry of the equipment. In this case, optimal working conditions were: 2.7 kbar; *OD*_0_, 60; *N*, three passes.

Optical density decrease, *TPR*, and *APAR* can be stated as general disruption indicators since similar release pattern is expected for other intracellular components of interest. The methodology described to evaluate the efficacy of a disruption procedure or equipment can be applied to optimize these processes, which aim reliable quantification of intracellular cell components. From the results presented in this work, one can conclude that using non-optimized cell disruption procedures can introduce important error in the assays and processes derived from it. Therefore, the quantification of intracellular components, such proteins, metabolites, and other cellular elements of interest, may not be accurate. In addition, the important decrease in recovery yields due to use of non-optimized cell disruption procedures may affect dramatically the efficiency of a bioprocess.

This article demonstrates the importance of the efficiency in cell disruption procedures for research studies derived from the quantification of intracellular components. Furthermore, the contribution is expected to have a big interest in bioprocesses for the recovery of the intracellular components of different cell factories, such recombinant or homologous proteins and enzymes, metabolites, and others.

## Conflict of Interest Statement

The authors declare that the research was conducted in the absence of any commercial or financial relationships that could be construed as a potential conflict of interest.

## Abbreviations

APAR, alkaline phosphatase activity released (AU·OD^−1^·L^−1^); DoE, design of experiments; HPH, high-pressure homogenization; KPI, key performance indicator; *N*, number of passes; OD, optical density; OD_0_, initial OD; ODD, OD decrease, %; RSD, relative SD, %; RSM, response surface methodology; SEE, SE of the estimate, %; TPR total protein released (mg·OD^−1^·L^−1^).
